# Barnyard millet (*Echinochloa* species): an underutilized nutritional powerhouse with emerging nutraceutical benefits

**DOI:** 10.3389/fpls.2026.1794657

**Published:** 2026-07-21

**Authors:** Minnu Sasi, Veda Krishnan, Ramesh M. Naik, Chandrika Das, Damodar Reddy Dendi, Aditya Nath Jha, Awadhesh K. Pal, S. R. Arpitha, Aditi Raj, Akanksha Sharma, Kashish Shrestha, Karan

**Affiliations:** 1 Department of Crop Improvement, ICAR- Central Plantation Crops Research Institute, Kasargod, Kerala, India; 2 Research Systems Management Division, ICAR-National Academy of Agricultural Research Management, Hyderabad, India; 3 Division of Biochemistry, ICAR-Indian Agricultural Research Institute, New Delhi, India; 4 Department of Biochemistry, North Bengal Regional R&D Centre, Tea Research Association, Nagrakata, West Bengal, India; 5 Department of Agriculture, Dev Bhoomi Uttarakhand University, Naugaon, Uttarakhand, India; 6 Department of Plant Physiology & Biochemistry, Bihar Agricultural University, Sabour, India

**Keywords:** arabinoxylan, bioethanol, bioprocessing, climate-resilience, nutri-cereal, plant bioactive, prebiotics

## Abstract

The impact of climate change presents an opportunity for orphan crops like millet to contribute to sustainable food systems. A prime example of an orphan crop with special traits and the potential to develop climate-smart agriculture is Barnyard millet (BYM). Alkaloids, steroids, polysaccharides, glycosides, tannins, phenols, dietary lignans and flavonoids are just a few of the antioxidants that are abundant in BYM. BYM’s antioxidant potential, prebiotic status, anti-inflammatory, hypoglycemic, antibacterial and anticancerous properties help it to combat a myriad of diseases. The antinutritional compounds present in BYM such phytic acid, tannins, polyphenols, and amylase inhibitors limit the absorption of minerals because they form complexes with dietary minerals like calcium, zinc, magnesium, and iron and make them inaccessible for absorption. Various processing methods like dehulling, soaking, heating, gamma irradiation, cold plasma processing, fermentation might enhance the nutritional and technological functional qualities of BYM. The bioactives in BYM can improve their bioavailability, *in vitro* digestibility, efficiency, structural modification and stability by biological processing techniques such as germination and fermentation employing microbial strains. BYM straw’s high cellulose and hemicellulose percentage makes C5 and C6 sugars accessible for bioconversion into bioethanol.

## 1 Introduction

Our planet’s population is anticipated to reach ∼10 billion by the year 2050 (Ghosh et al., 2024). Food security is a major challenge across the globe, especially in the least developed countries. The COVID-19 pandemic, population hikes, and climate change have all brought attention to the need to incorporate low-input crops that can adapt to broader changes in the environment. One feasible remedy is to grow “orphan crops,” which are equally nutritious and diverse. The performance of orphan crops is better than that of major crops under the current scenario of a global climatic crisis on account of their unique attributes, including adaptation on marginal lands, less water requirement, enhanced biotic and abiotic stress tolerance, and outstanding nutritive value (Yang et al., 2023). Among the orphan crops, millets play a pivotal role in the poor and malnourished population’s nutritional security and can be a game-changer in the long term (Kumar et al., 2023). They also act as a source of income generation for poor and marginal farmers in developing countries (Dekka et al., 2023).

Millets are small-seeded nutri-cereals that belong to the family Poaceae and are well known for their ability to grow in dry land. Millets are termed “yesterday’s coarse grains and today’s nutri-cereals” (Gowda et al., 2022), as they are exceptional nutritional crops with a significant quantity of proteins, vitamins such as niacin and riboflavin, essential fatty acids, essential amino acids, and minerals like zinc, iron, potassium, calcium, and magnesium. Millets contain a high proportion of dietary fiber, which gives a low glycemic index and helps to maintain a low blood glucose content. Owing to the presence of nutraceutical components, millet consumption is good for lowering cholesterol, obesity, gastrointestinal abnormalities, modulating insulin resistance, and inflammation in the gut (Muskan et al., 2025). Patients with diabetes mellitus and celiac disease can use millet in their diets because they are gluten-free (Gami et al., 2026).

The share of India in millet cultivation area is between 85% and 80% in production in Asia (Tonapi and Bhat, 2025), and India is the largest producer of millet in the world currently. According to the market analysis, the estimated value of the millet market at the global level reached $11.53 billion in 2024, and along with that, by 2029, the estimated value is expected to reach $14.53 billion, growing at a compound annual growth rate percentage of 4.60% (Baduni et al., 2024; Yadav et al., 2025).

Millet crops consist of major millets like sorghum, pearl millet, and finger millet, and minor millets like foxtail, little, kodo, proso, and barnyard millet (BYM) (Sobti et al., 2023). BYM (*Echinochloa* species consisting of *Echinochloa esculenta*, *Echinochloa frumentacea*, and *Echinochloa utilis*) is one of the most prominent among the minor millets. BYM is grown naturally in the Uttarakhand and Tamil Nadu states of India and has recently shown a boon in production. Over the previous three years, the BYM production area in India has increased to 0.147 m/ha and has produced 0.148 million tons with an average productivity of 1035 kg/ha (Bhatt et al., 2023). The optimum day and night temperatures needed for its growth range between 26-35 °C and 15-23 °C, respectively. BYM is a rapidly growing annual crop largely grown for human consumption and is widely accepted as a voluminous fodder crop in the Himalayan region owing to its highly palatable nature and broad leaves, which are helpful in hay or silage preparation (Sood et al., 2015).

This article has been formulated to evaluate BYM’s nutritional makeup, bioactive substances, and functional attributes critically, with a focus on how it can improve dietary quality, metabolic health, and malnutrition. Also, the article examines how different processing techniques affect the improvement of techno-functional and sensory qualities, the decrease of anti-nutritional elements, the increase of bioavailability, consumer acceptability while figuring out how to improve its widespread use in sustainable food systems.

## 2 BYM- a climate resilient nutri-cereal

Millets acquire diverse morpho-physiological, molecular, and biochemical characteristics to offer increased capacity to tolerate climate change. Its quick growth habit and adaptable phenology (short growing season) let it thrive on sandy, loamy, and generally poor soils. A wide genetic background in germplasm collections and a variety of landraces that function in various moisture and temperature conditions are the sources of this adaptability (Yadav et al., 2025). Compared to many staples, BYM requires less water and nutrients. The short life cycle of millets, i.e., ∼10–12 weeks, can be considered an adaptive mechanism for the mitigation of stress, while other major cereal crops have a life cycle of 20–24 weeks (Vadez et al., 2024). BYM is a C4 plant with a hatch and slack pathway, which is extra advantageous for the plant to survive in areas with high temperatures and low moisture (Babele et al., 2022). Delving into its mechanism and adaptations, during the Hatch and Slack pathway, due to Kranz anatomy, the light-dependent reactions take place in mesophyll photosynthetic cells, but the Calvin cycle takes place in bundle-sheath cells to enhance the amount of CO_2_ (von Caemmerer and Furbank, 2003). This spatial separation prevents photorespiration (around 80%), which is a wasteful process in terms of energy that occurs when bundle sheath cells come into contact with O_2_. Therefore, the photosynthetic rate in BYM is very high at a warm temperature and results in excessive water and nitrogen use efficiency, which is ∼1.6- to 3.5-fold more than the C3 mechanism.

Furthermore, C4 photosynthesis imparts secondary advantages like better growth and ecological performance, improved flexible allocation patterns of biomass, and reduced hydraulic conductivity per unit leaf area (Babele et al., 2022). Also, BYM can regulate its membrane dynamics to enhance water permeability and achieve higher water status during drought stress. Morphological adaptations to mitigate drought stress include high leaf tensile strength, thickened cell walls, and lengthy and dense root systems (Chellapilla et al., 2022). Diverse molecular and biochemical mechanisms, including reactive oxygen species (ROS) generation, can enhance the ROS-scavenging enzymes (superoxide dismutase, ascorbate peroxidase, monodehydroascorbate reductase, dehydroascorbate reductase, glutathione synthetase, catalases) and other proteins involved in stress tolerance (Singh et al., 2022). Also, BYM is reported to be recommended as a natural phytoextractor of heavy metals (lead, cadmium, and chromium) in contaminated soils and sodic soils (Renganathan et al., 2020). Farmers may cultivate it with minimal external input and minimize production costs due to its short cycle and modest fertilizer uptake (Hasan et al., 2025).

### 2.1 Nutritional composition and phytochemicals

Nutritionally, BYM is an exceptional crop compared to other major and minor millets. The optimal concentration of total carbohydrates in BYM ranges between 52 and 62.5 g/100 g fresh weight (Saleh et al., 2013), which is the lowest of other major and minor millets and makes it the least caloric dense. In BYM, the amount of dietary fiber is greater than any other nutrient cereal and varies between 8.0 and 16.4 g/100 g with soluble (5.67 g/100 g) and insoluble (10.14 g/100 g) fractions (Ugare et al., 2014). The amount of protein in BYM varies from 11.3-12.8%, which is remarkably high as compared to other millets. The essential amino acids reported in BYM include lysine, methionine, threonine, isoleucine, leucine, histidine, and tryptophan, and the non-essential amino acids present include aspartic acid, glutamic acid, arginine, alanine, cysteine, glycine, and proline (Sharma N. et al., 2022). The amount of lipid in BYM is approximately 5.31%, which is lower than other millets. It is often recognized that BYM is a good source of oleic, linoleic, and palmitic acids-three important beneficial fatty acids. Also, the iron content of 15.7-18.8 mg/100 g of raw grains makes it the richest of all nutri-cereals (Renganathan et al., 2020). The calcium content of BYM is reported to be approximately 24.16 mg/100g raw grains; meanwhile, the amount of magnesium is 38.21 mg/100g raw grains of BYM. **Table 1** presents the macro and micronutrient composition of BYM in detail. It is also an excellent source of potassium (299 mg/100 g of raw grains), which controls vasodilation, thus protecting us from cardiovascular disorders through blood pressure reduction (NazniDevi et al., 2016). BYM is also an excellent source of antioxidants such as alkaloids, steroids, glycosides, tannins, phenols, dietary lignans, and flavonoids (Padhiyar et al., 2024). Total phenolic content (TPC) and flavonoid contents in BYM were reported to be approximately 30.03 mg (Gallic Acid Equivalent-GAE)/100g and 30.04 mg RU/g, respectively (Sharma et al., 2016). The flavonoid 3,7-dimethyl quercetin and polyphenolic components like formononetin, luteolin, apigenin, catechin, kaempferol, and isorhamnetin are also reported in BYM (Dutta et al., 2023). **Table 2** presents a detailed **c**omparison of nutritional composition between different types of millets with BYM.

**Table 1:** Macro and micronutrient composition of BNM.

Category	Content	Subcategory	Content	References
Macronutrients				
Carbohydrates	68.8 %	Starch	51.5-59.5 %	Veena, 2005
		Amylose	104 g/kg	Vali Pasha et al., 2018
Dietary fiber	12.6%	Crude fiber	13.6 g/100 g	Saleh et al., 2013
		Total dietary fiber	252 g/kg	Vali Pasha et al., 2018
		Insoluble dietary fiber	6.1–10.5%	Veena, 2005
		Soluble dietary fiber	3.5–4.6%	
Protein	10.5%	Prolamin	14.3-20.9%	Serna-Saldivar and Ramirez, 2019
		Albumin and globulins	11.3-17.2	
		Glutelins	45.2-63.5	
Amino acids		Isoleucine	725 mg/g	FAO, 2007
		Leucine	725 mg/g	
		Valine	388 mg/g	
		Methionine	133 mg/g	
		cystine	175 mg/g	
		Threonine	231 mg/g	
		Histidine	2.0 mg/100g	Renganathan et al., 2020
		Lysine	106 mg/g	FAO, 2007
		Phenyl alanine	362 mg/g	
		Tyrosine	150 mg/g	
		Tryptophan	63 mg/g	
Lipids (%)	2.5-3.6% (Indira and Naik, 1971)	Neutral lipid	85.5 %	Sridhar and Lakshminarayana, 1992
		Glycolipid	9%	
		Phospholipids	5.5 %	
	Fatty acid composition in Neutral lipid	Palmitic acid (16:0)	17.4 %	
		Palmitoleic acid (16:1)	0.4 %	
		Stearic acid (18:0)	4.3 %	
		Oleic acid (18:1)	27.6 %	
		Linoleic acid (18:2)	48.1 %	
		Linolenic acid (18:3)	1.5 %	
		Arachidonic acid (20:0)	0.3 %	
		Arachidic acid (20:4)	0.1 %	
		Behenic acid (22:0)	0.2 %	
		Erucic acid (22:1)	0.1 %	
Ash (g/100g)			4.7-5.0 g/100 g	Renganathan et al., 2020
Minerals				
Major Minerals		Phosphorus (P)	340 mg/100 g dry matter	Muthamilarasan et al., 2016
		Potassium (K)	7.34–7.92 g/kg	Sharma et al., 2021
		Sodium (Na)	0.68–0.69 g/kg	
		Calcium (Ca)	22 mg/100 g dry matter	Muthamilarasan et al., 2016
		Magnesium (Mg)	86.2 mg/100 g dry matter	
Trace minerals		Iron (Fe)	18.6 mg/100 g dry matter	Muthamilarasan et al., 2016
		Zinc (Zn)	4.9 mg/100 g dry matter	
		Manganese (Mn)	0.7 mg/100 g dry matter	
		Copper (Cu)	0.6 mg/100 g dry matter	
		Cromium (Cr)	0.14 mg/100 g dry matter	
Micronutrients				
Water soluble Vitamins		Vitamin B1 (Thiamin)	0.4 mg/100 g	Renganathan et al., 2020
		Vitamin B2 (Riboflavin)	0.1 mg/100 g	
		Vitamin B3( Niacin)	4.2 mg/100 g	
		Vitamin C (Ascorbic acid)	44.05- 54.15 μg/g	Panwar et al., 2016
Fat soluble vitamin		α-tochopherol	23.6-30.9 (μg/ g)	
		Total carotenoids	36.7-50.8 (μg/ g)	
Bioactive compounds		GABA	11.5-12.3	Renganathan et al., 2020
		*β*-glucan	5.0-6.0	
		Total phenol	20.3 - 27.80 mg GAE/ g	Panwar et al., 2016
		Flavonoids	3.12- 5.26 mg QE/g	
		Tannins	3.25- 3.96 mg/g	
		Saponins	8.38- 10.26 mg diosgenin eq/g,	
		Orthodihydroxyphenol	107.14- 130.71 μg catechol/g	
Anti-nutritional factors		Phytic acid	3.30- 3.70 mg/g	Panwar et al., 2016
		Trypsin inhibitor	7.85- 8.37 (%)	

**Table 2:** Comparison of nutritional composition between different types of millets with BYM.

Name of the millet	Scientific name	Common name	Protein (g)	Fat (g)	Ash (g)	Crude Fiber (g)	Carbohydrate (g)	Energy (kcal)
Sorghum	*Sorghum bicolor*	Jowar, milo, guinea corn, kaoliang, dura, shallu	10.4	3.1	1.6	2.0	70.7	329
Pearl Millet	*Pennisetum glaucum*	Bajra, gero, arum, bulrush millet, milheto, dark millet, cattail millet	11.8	4.8	2.2	2.3	67.0	363
Finger Millet	*Eleusine coracana*	Ragi, african millet, red millet, caracan millet, Koracan, dagusa	7.3	1.3	2.7	3.6	72.0	336
Foxtail Millet	*Setariaitalica*	Italian millet, german millet, chinese millet, hungarian millet, green millet	12.3	4.3	3.3	8.0	60.9	351
Proso Millet	*Panicum miliaceum*	Broomcorn millet, common millet, broomtail millet, hog millet, white millet	12.5	3.1	1.9	7.2	70.4	364
**Barnyard Millet**	*Echinochloa utilis*	Sawa millet, japanese barnyard millet, indian barnyard millet, kodisama, bhagar, burgu millet	**6.2**	**2.2**	**4.4**	**9.8**	**65.5**	**300**
Little Millet	*Panicum sumatrense*	Samalu, samai	7.7	4.7	1.5	7.6	67.0	329
Kodo Millet	*Paspalum scrobiculatum*	Varagu, varigalu	8.3	1.4	2.6	9.0	65.9	353
Teff	*Eragrostis tef*	Tahf, lovegrass, williams’ lovegrass, xaafii, taffi, chimanganga	11.0	2.5	2.8	3.0	70.2	357
Coix	*Coixlacryma-jobi*	Job's Tears, Adlay, Chinese Pearl Barley	12.8	3.8	1.8	1.3	66.5	356
Browntop Millet	*Brachiariaramosa*	Korale, signal Grass	11.5	3.7	2.1	10.0	58.0	332
Rice	*Oryza sativa*	--	6.8	0.5	0.6	0.2	78.2	362
Wheat	*Triticum aestivum*	--	11.8	1.5	1.5	1.2	71.2	348

BYM has high crude fibre and highest ash content compared to other millets.

### 2.2 BYM starch and its properties

The higher purity and yield value of BYM starch indicate its potential for use in commercial starch production. BYM has a total starch content that varies from 51.5% to 62% (Renganathan et al., 2020). Granules of BYM starch typically have an A-type crystallinity in different shapes, from spherical to polygonal (6.46–12.23 μm) (Verma et al., 2018). BYM starch was reported to have an amylose percentage between 17.03% and 23.30% (Bangar et al., 2021). It falls within the intermediate to high amylose starch range (20%–25%), and it can be used as one of the main components of formulations including resistant starches (RSs) (Wu et al., 2014). Low glycemic index and delayed starch digestion are due to the starch composition of BYM, which is mostly composed of higher amylose than amylopectin content. These characteristics assist in controlling postprandial blood glucose levels (Lal et al., 2021). The higher solubility and swelling capacity of starches make them useful for use as stabilizers and binders in food items (Mahmood et al., 2017). BYM was found to have the highest swelling power, with a reported value of 18.83 g/g (Bangar et al., 2021). The viscosity parameters measured in BYM were determined to be final (3454 ± 21cP), peak (2986 ± 20cP), breakdown (1437 ± 16cP), trough (1548 ± 15cP), and setback (1903 ± 21cP) viscosities, respectively (Bangar et al., 2021). The peak viscosities displayed by BYM starches suggest their potential use in a variety of culinary applications, including puddings, salad dressings, and desserts.

### 2.3 Phenolics and flavonoids

It was found that the bound form of BYM has a significantly higher phenolic content than the free form. Sharma et al. (2016) reported that the total phenolic content was 30.01 ± 0.3, with free phenolic concentrations of 11.46 ± 0.15 and bound phenolic concentrations of 17.55 ± 0.16. Similarly, bound fractions dominated the flavonoid concentration of BYM. The overall flavonoid content was 29.02 ± 0.15, with free flavonoids accounting for 9.48 ± 0.06 and bound flavonoids for 19.54 ± 0.09. Overall, these findings show that the majority of BYM total bioactive chemicals are bound phenolics and flavonoids, especially in the form of arabinoxylans, underscoring the grain’s potential as a functional diet strong in antioxidant components.

### 2.4 Phytosterols

Gas Chromatography-Mass Spectrometry analysis of methanolic extract BYM seed extract showed a myriad of bioactive compounds in BYM (Sharma et al., 2016). Benzoic acid, 4-ethoxyethyl ester, 9H-fluoren-9-one, eicosane, and xanthone were the main compounds found in BYM genotype VL Madira 29. However, in VL 232 genotype, the major compounds were caprolactam, campesterol, stigmasterol, γ-sitosterol, (Z)-9,17-octadecadienal, 4-((1E)-3-hydroxy-1-propenyl)-2-methoxyphenol, hexadecanoic acid. The steryl ferulates, a specific conjugated phytosterols in BYM, varies significantly depending on its processing and place of origin. The steryl ferulates content of whole grain was 4.78 ± 0.57 in samples from Iwate, Japan, and 4.88 ± 0.40 in samples from Aomori, Japan. Conversely, steryl ferulates were significantly reduced in dehulled BYM, with values falling to 1.30 ± 0.22 for samples from Iwate and 1.65 ± 0.27 for those from Aomori. Overall, these findings show that steryl ferulates are mostly found in the outer grain layers and that processing of BYM significantly reduces the amount of these bioactive substances (Tsuzuki et al., 2018).

### 2.5 Arabinoxylans

BYM has been found to contain non-cellulose and non-starch polysaccharides in the form of phenolic acid-bound arabinoxylans (Bijalwan et al., 2016). According to Wang J. et al. (2022), feruloylated arabinoxylans typically activate *Toll-Like Receptors* via the Nuclear Factor kappa-light-chain-enhancer of activated B cells (NFĸB), Extracellular Signal-Regulated Kinase (ERK), Activator Protein-1 (AP-1), and Mitogen-Activated Protein Kinase (MAPK) pathways. This results in the production of a variety of proinflammatory mediators, including Inducible Nitric Oxide Synthase (iNOS), Cyclooxygenase-2 (COX-2), (Tumor Necrosis Factor-alpha) TNF-α, Interleukin-1 beta (IL-1β), Interleukin-6 (IL-6), and others. The study conducted by Srinivasan et al. (2020) assessed the immunostimulatory potential of phenolic acid-bound arabinoxylan from BYM and foxtail millets by examining their impact on the release of nitric oxide (NO), ROS, and cytokines (TNF-α, IL-1β, and IL-6) in RAW 264.7 murine macrophage cells. According to the study, BYM has a greater influence on macrophage activation than foxtail millet because it contains bound phenolic acids with a composition that includes ferulic acid, p-coumaric acid, and caffeic acid with highly branched arabinoxylans. Due to the presence of ferulic acid, feruloyl arabinoxylans showed extremely robust antioxidant activity in a previous study on finger millet too (Subba Rao and Muralikrishna, 2006). In peritoneal mice, it was also observed that the arabinoxylans coupled with ferulic acid produced from finger millet bran demonstrated an immunostimulation effect (Savitha Prashanth et al., 2015). Arabinoxylans also have prebiotic effects when they interact with gut flora (Jacob et al., 2024; Gonzalez-Alonso et al., 2026).

## 3 Nutraceutical value of BYM

On account of the presence of a plethora of bioactive compounds, BYM has antioxidant, antimicrobial, anti-diabetic, anti-inflammatory, and anti-carcinogenic nutraceutical properties that mitigate the risk of various diseases (Renganathan et al., 2020).

### 3.1 Antioxidant potential

Polyphenolic compounds, including flavonoids (isoflavones), phenolic acids, stilbenes, and proanthocyanidins, are remarkable radical scavengers and are identified to play a crucial role in the prevention of such diseases and stress conditions (Sasi et al., 2021). N-(p-coumaroyl) serotonin, luteolin, quercetin, tricin, triacetin, ferulic acid, and some lipophilic antioxidants, comprising vitamin E and carotenoids, are some of the compounds isolated from BYM with antioxidant activity (Watanabe, 1999). The study conducted by Panwar et al. (2016) showed that the total polyphenol content in BYM varieties ranged from 20.32 (VL172) to 27.82 (VL21) mg GAE/g, which is reported to be higher than in the seeds of finger millet (10.25 (VL315) to 12.56 (VL146) mg GAE/g. In the same study, the ortho-dihydroxy phenol content in seeds of BYM varieties ranged from 106.74 to 131.61 μg catechol/g, whereas in finger millet varieties it ranged from 52.87 to 65.29 μg catechol/g. Hence, it was revealed that the amount of ortho-dihydroxy phenol, a significant class of plant polyphenolics in BYM, is approximately two times higher than that of finger millet in BYM.

Among the flavonoids, the 3′,4′-dihydroxy structure (catechol structure) is a requisite for strong antioxidant activity. The concentration of flavonoids in BYM seeds ranged from 3.11 to 5.27 mg Quercetin Equivalent/g compared to the seeds of finger millet, ranging from 3.34 to 4.16 mg QE/g (Panwar et al., 2016). Another important class of antioxidants is carotenoids and α-tocopherols, which are predominant peroxyl radical scavengers that aid in securing polyunsaturated fatty acids (PUFAs) present in the membrane phospholipids (Duan et al., 2024). The total carotenoid concentration in BYM seeds ranged from 37.12 to 51.78 µg/g, which is twofold higher compared to finger millet. Meanwhile, α-tocopherol content in BYM varied from 24.61 to 31.93 µg/g. In another study to estimate the nutritional and physicochemical properties of 13 varieties of BYM, the ethanolic extract of variety K141285 exhibited potent antioxidant activity against 2,2′-Azino-bis(3-ethylbenzothiazoline-6-sulfonic acid) (ABTS) (half maximal effective concentration (EC50): 74.9 ± 1.86 mg/mL) and 2,2-Diphenyl-1-picrylhydrazyl (DPPH) (EC50: 87.6 ± 2.26 mg/mL) on account of the presence of a higher number of phenolic compounds (Kim et al., 2011). BYM is also reported to have higher antioxidant and detoxification enzymes such as acid phosphatase, α-galactosidase, and α-amylase inhibitors compared to other millet crops (Kumaraguru et al., 2024; Srivastava et al., 2024). These results propose that the consumption of BYM products with these bioactive compounds contributing to the antioxidant effect can ameliorate the health of the consumer.

### 3.2 Antimicrobial potential

Antimicrobial activity is also a distinguished therapeutic attribute exhibited by BYM extracts. BYM (*E. esculenta*) methanolic extract showed antimutagenicity against 3-(5-nitro-2-furyl) acrylic acid in *Salmonella typhimurium* strains because of the presence of abundant antioxidants, which play a prominent role in H_2_O_2_ scavenging (Mosovska et al., 2010). Also, a distinctive antimicrobial peptide, namely, EcAMP1, with a broad spectrum of antifungal properties against fungal pathogens such as *Alternaria*, *Botrytis*, *Fusarium*, and *Trichoderma*, was recognized in the seeds of BYM (*E. crusgalli*). In another study, the seed protein extracts of minor millets, including Japanese BYM, pearl millet, sorghum, foxtail millet, samai millet, and proso millet, were checked *in-vitro* for their capacity to hinder the survival of *Rhizoctonia solani*, *Macrophomina phaseolina*, and *Fusarium oxysporum*. The Western blot analysis also showed the existence of 23-kDa thaumatin-like proteins (TLPs) in the grains of pearl millet, sorghum, and Japanese BYM (Radhajeyalakshmi et al., 2003). TLPs are associated with antifungal, glucanase, and xylanase inhibition activities and therefore play an important role in host defense, stress tolerance, and cell signaling (Feng et al., 2024).

### 3.3 Antidiabetic potential

Type 2 diabetes (T2D) is one of the most common and chronic diseases that affects our body’s metabolism. BYM is an exceptional source of polyphenols such as apigenin, kaempferol, formononetin, dihydroquercetin, catechin, p-coumaroyl serotonin, feruloyl serotonin, and luteolin, which can effectively hinder α-glucosidase and pancreatic α-amylase activities, enzymes that hydrolyze non-absorbable carbohydrates into glucose (Ofosu et al., 2020). Ethanolic extracts of BYM showed the highest inhibition of the α-glucosidase enzyme with half maximal inhibitory concentration (IC50) values of 21.91µg/mL, which was remarkably effective in comparison with the generally prescribed standard drug, acarbose IC50 value (58.84µg/mL) (Ofosu et al., 2020). In the same study, Italian millet showed the lowest α-glucosidase inhibition with IC50 values of 498.76 µg/mL (Wang et al., 2022). This inhibitory effect on α-glucosidase by BYM also reduced the generation of advanced glycation end-products, a serious biochemical consequence of the onset and progression of the disease. Also, polyphenols could attenuate diabetes and postprandial hyperglycemia by initiating the adenosine monophosphate (AMP)-activated protein kinase pathway (Sears and Saha, 2022). Hence, polyphenols in BYM can remarkably alleviate glucose metabolism by regulating glucose homeostasis. High amylose retrogradation is a property of starch present in BYM that aids in the generation of a high concentration of resistant starch that helps in improving your body’s potential to respond to insulin. The higher the ratio of carbohydrates to crude fiber, the slower the movement of sugars into the blood; thus, consumption of BYM regulates the blood sugar level (Renganathan et al., 2020). Also, the Japanese BYM bran diet ameliorated diabetic polyuria in rats. The HbA1c levels were also reduced after the diabetic rats were fed with the BYM bran diet on account of the enhanced expression of heme oxygenase-1, an antioxidative enzyme present in the liver (Ito et al., 2022). These results propose that the BYM bran diet lowers an acute increase in blood glucose levels post-food consumption and has immense ameliorative and antioxidative attributes during diabetes (Wang H. et al., 2022).

### 3.4 Anti-inflammatory potential

A study was conducted to estimate the anti-inflammatory potential of BYM (*E. crusgalli* var. frumentacea) grains against lipopolysaccharide -induced inflammatory actions in the mouse macrophage cell line RAW 264.7. In the study, a higher concentration of anti-inflammatory phenolic compounds, including kaempferol (9.18 μg/mg), biochanin A (2.23 μg/mg), and formononetin (1.53 μg/mg), were detected after consequent fractionation with methylene chloride. Also, some of the minor phenolic compounds that have anti-inflammatory activity, like hesperidin (0.34 μg/mg), naringin (0.57 μg/mg), and protocatechuic acid (0.35 μg/mg), were detected in the methyl chloride fraction. The aforementioned compounds observed in the methyl chloride fraction of the ethanolic extract of BYM could repress the lipopolysaccharide-induced nuclear translocation of cytosolic NF-kB and also the lipopolysaccharide-induced stimulation of MAPKs, including ERK, Jun N-terminal Kinase (JNK), and p38MAPK (Lee et al., 2014). Other prominent compounds of BYM that have anti-inflammatory activity are bioactive peptides (Dutta et al., 2023; Semwal et al., 2024).

### 3.5 Anticancerous potential

A study was carried out to manifest the anticancerous attributes of specific compounds present in BYM grains against human cancer cell lines, including MCF-7 (breast carcinoma), HCT-116 (colon carcinoma), HELA (cervical carcinoma), and HEPG-2 (liver carcinoma) (El Molla et al., 2016). The cytotoxic potential of BYM grain alcoholic extracts at a concentration of 50 g/mL revealed IC_50_ values of 13.0 ± 0.12, 11.3 ± 0.10, 18.8 ± 0.13, and 14.3 ± 0.12 g/mL against HELA, HCT-116, MCF7, and HEPG-2 cells, respectively. The chloroform and ethyl acetate extracts displayed remarkably high tumorous activities against the HCT-116 cell line with IC_50_ values of 3.9 ± 0.01 and 4.3 ± 0.04 g/mL, respectively, which was proportionate with the standard anticancerous drug doxorubicin (IC_50_ 4.6 ± 0.54 g/mL). The purification of cytotoxic compounds from these fractions using nuclear magnetic spectroscopy identified 5,7-dihydroxy-3,4,5-trimethoxy flavone, 5,7,4-trihydroxy-3,5-dimethoxy flavone (tricin), quercetin, flavone, apigenin-8-C-sophoroside, and quercetin-3-O-glucoside in BYM grains. The study also proved that methoxylated compounds showed predominantly higher cytotoxic activities than phenolic compounds and the anticancer drug doxorubicin (El Molla et al., 2016). Using the MCF-7 cell lines, the antiproliferative activity of BYM was assessed (Ramadoss and Sivalingam, 2020). The results showed that MCF-7 cells proliferated after 48 hours of treatment with a 1000 μg/mL concentration of BYM extract. The study demonstrated cell arrest in the G_0_/G_1_ phase and multiplied the number of apoptotic cells in the sub-G_0_ phase in a dose-dependent manner.

### 3.6 Prebiotic potential

The prebiotic activity score of a substrate can be measured in terms of the adequate growth and survival of probiotic bacteria in comparison to when they are supplemented with glucose. In a study, a millet functional beverage comprising BYM (6.9 g), foxtail (8.9 g), and kodo (7.9 g) millets was fortified with well-known prebiotics such as fructooligosaccharides (1.3 g/100 g w/v) and galactooligosaccharides (1.5 g/100 g), which were shown to improve the sensory properties. The prebiotic activity score of millet functional beverages with fructooligosaccharides was 1.55 ± 0.31 and with galactooligosaccharides was 1.46 ± 0.26. Surprisingly, the prebiotic activity score of a millet functional beverage without any prebiotic was observed to be 0.47 ± 0.23. From this observation, it can be accepted that the control millet functional beverage without any additives also showed prebiotic activity due to the presence of adequate dietary fiber and will promote the growth of selective probiotic microorganisms (Arya and Shakya, 2021). Millet arabinoxylans have been identified as having prebiotic potential due to their capacity to influence gut flora (Chen et al., 2019).

Broadly, all these aforementioned attributes of BYM make it an appropriate cereal for consumers’ nutritional security. Certain *in-vitro* studies and human clinical trials showing health-promoting effects of BYM have been included in **Table 3**.

**Table 3:** *In-vitro* studies and human clinical trials showing health-promoting effects of BYM.

Sl No	Type of study	BNM food dosage	Health-associated effect	Reference
1	High-fat diet consisting barnyard millet protein was fed to Type-II diabetic mice for 3 weeks	63.2 g of barnyard millet protein in 100 g of diet	***Anti-diabetic effects*** Decreased the levels of glucose and ratio of triglyceride/High-Density Lipoprotein cholesterol which are surrogate markers of insulin resistance	Nishizawa, 2009
2	BNM grains with low glycemic index both dehulled (50.0) and dehulled and heat treated (41.7) was fed for 28 days to healthy participants between 38-40 years of age	10.6% protein 3.7% fat, 68.9% carbohydrate and 397 kcal/100 g energy	***Anti-cholesterol effects*** The feeding intervention exhibited a remarkable decrease in glucose (138.2 to 131.1 mg/ dl), LDL-C (from 168.7 to 162.8 mg/dl), VLDL-C (from 25.0 to 22.2 mg/dl), ratio of TC: HDL (from 4.8 to 4.7) and LDL: HDL (from 3.3 to 3.2)	Ugare et al., 2014
3	Colon cancer HT-29 cells were treated with bioactive compound extracted from barnyard millet with chemical structure similar to the phenolic aldehyde-Vanillin [4-Hydroxy-3-methoxybenzaldehyde]	250, 500, 750, and 1,000 mg/ml concentration for 48 h	***Anti-cancerous effects*** HT-29 cell proliferation and apoptosis induction was observed. Flow cytometry results exhibited cell arrest in the G0/G1 phase and augmentation in the apoptotic cells in sub G0 phase.	Ramadoss and Sivalingam, 2020
4	The effect of (Echinochlorins A-C; 1, 8, 9) isolated form the methylene chloride and ethyl acetate extracts of Echinochloa utilis on nitric oxide (NO) production in lipopolysaccharide (LPS)-activated murine macrophage RAW 264.7 cells	50, 100, 250 µg/ml concentration	***Anti-inflammatory effects*** Echinochlorins A-C; 1, 8, 9 possessed to have NO inhibitory activity against lipopolysaccharide (LPS)-induced NO release with IC_50_ values of 35.04, 4.70, and 20.77µM, respectively.	Woo et al., 2015
5	Feeding intervention of millet-based diet (barnyard millet, finger millet and a mixture of both) from a period of 21 days to 2 years	Millet foods were consumed either in the form of biscuits, burfi (sweet), porridge, buns, roti (flatbread), dumpling	***Anti-obesity effects*** Upon consumption of millet-based meals, there was a 7.1% decrease in the body mass index (BMI) and 5.1% decrease in systolic and diastolic blood pressure	Anitha et al., 2021

## 4 BYM- antinutritional factors

Antinutritional factors like tannins and phytic acids have a detrimental effect on *in vitro* protein digestibility and cause damage to the gastrointestinal tract, but in BYM, tannin compounds accumulate in the bran. The total tannin content, oxalate content, and trypsin inhibitor activity reported in the grains of a BYM variety *E. frumentacea* BMVL-29 were 0.302 g/100g, 0.03 g/100g, and 32.96 g/100g, respectively, which is considered to be less than the threshold level and would not cause any health hazard (Gupta and Shrivastava, 2015). In another study, tannins in the seeds varied from 3.27 to 3.93 mg/g and saponins from 8.37 to 10.25 mg diosgenin equivalent/g (Panwar et al., 2016). The initial phytic acid content in BYM variant VL-172 was reported to be approximately 150 mg/100 g before it was reduced through cold plasma processing (Charu et al., 2024). Millets also contain goitrogens, which, especially in high concentrations, can interfere with the thyroid’s ability to absorb iodine and cause thyroid dysfunction (Siroha and Bangar, 2024). Reductions of these antinutritional factors through processing are a major requirement to procure the absolute nutritive value of BYM.

## 5 Bioprocessing and value addition of BYM

Despite its nutritional benefits and adaptive traits, BYM has remained an underutilized crop due to the anti-nutritional factors and sensory factors. Studies have demonstrated that conventional processing techniques like dehulling, soaking, and cooking, biological processing methods like germination and fermentation, and physical processing like gamma irradiation, high-pressure treatments, and cold plasma processing can effectively contribute to the enhancement of the techno-functional properties of BYM components. Overall, processing can augment the bioavailability, *in vitro* digestibility, efficiency, structure modification, and stability of proteins present in BYM.

### 5.1 Dehulling, soaking and germination

Dehulling, or decortication, is the process of peeling off the pericarp of millets (Mahajan et al., 2024). It is reported that dehulling BYM decreases the amount of phytates and tannins as these anti-nutrients are accumulated at the outer pericarp (Singh et al., 2024). Dehulling was identified to reduce the total phytic acid remarkably but also enhance the protein digestibility in BYM (Goel et al., 2021). In a study, a feeding intervention of 28 days was conducted to observe the effect of dehulled BYM grain, which showed a significant reduction in glucose (138.3 to 130.0 mg/dl), low-density lipoprotein cholesterol (LDL-C) (from 166.6 to 161.8 mg/dl), very low-density lipoprotein cholesterol (VLDL-C) (from 23.0 to 22.1 mg/dl), and the ratio of total cholesterol to high density lipoprotein cholesterol (HDL) (from 4.6 to 4.5) and LDL to HDL (from 3.1 to 3.0) in the experimental diabetic groups (Ugare et al., 2014).

Another conventional processing technique is the soaking of the grains, which is a very common household technique and is the easiest way to reduce the antinutrient content in millet. Soaking of BYM grains for 8 hours at 40°C for germination has increased the TPC, antioxidant activity, and total flavonoid content of BYM up to 45.66%, 78.98 mg (GAE)/100 g, and 37.01 mg/g. While soaking the BYM grains for 12 hours at 30°C for germination has further increased the TPC, antioxidant activity, and total flavonoid content of BYM up to 55.29%, 91.33 mg gallic acid equivalents (GAE)/100 g, and 42.23 mg/g (Sharma et al., 2016).

The process of germination is reported to bring noteworthy alterations in the biochemical, nutritional, and sensory profiles of cereal grains because of the breakdown of reserve materials for respiration and the generation of cell wall constituents and cellular components for the growing embryos (Dubey and Tripathy, 2024). Germination is reported to have a consequential influence on the TPC, antioxidant potential, dietary fiber content, γ-aminobutyric acid, and mineral concentration of BYM (Durairajan et al., 2024). In a study conducted by Sharma et al. (2016), the germinated BYM showed an increase in the amounts of hexadecenoic acid (5.57%), octadecadienoic acid (14.58%), 9,12-Octadecadienoic acid, ethyl ester (12.00%), c-sitosterol (5.00%), lupeol (5.19%), and campesterol (7.58%) in comparison to raw flour. All the aforementioned phenolic compounds are popular for their neuroprotective, anesthetic, antibacterial, antibiotic, antioxidant, anti-hyperlipidemic, and anti-tumor effects (Dekka et al., 2023; Daji et al., 2024). There was an inflation in total dietary fiber too because of the moderation of cell wall polysaccharides in germinating seeds, likely by altering the intactness of tissue histology and modulating the protein-carbohydrate interaction (Aanchal et al., 2024). The values of gamma-amino-butyric acid improved from 3.37 to 35.60 mg/100 g during germination in BYM, owing to the γ-decarboxylation of L-glutamic acid by the enzyme glutamate decarboxylase, which makes it a more useful food by raising its Gamma-aminobutyric acid (GABA) content and supporting positive effects on human health (Sharma et al., 2016). The concentration of free phenolic compounds like hydroxyl cinnamates (e.g., ferulic and p-coumaric acids) in grain cell walls, which are attached to non-starch polysaccharides, was found to increase in millet milk produced from BYM and other minor millets because of their release by the activity of cell wall-degrading enzymes like esterases (Shunmugapriya et al., 2020; Sunil et al., 2024). Germination is an active stage of metabolism in which antinutrients are lowered. Also, germination caused a remarkable hike in the iron, magnesium, calcium, and sodium contents of BYM flour owing to the activation of the phytase enzyme, which hydrolyses the phytate to inositol and free orthophosphate and releases minerals (Chethan Kumar et al., 2022). During soaking and germination, *in-vitro* protein digestibility rising from 72.13 to 76.61 g per 100 g of BYM flour (Dey et al., 2026). This is because the protein molecules were partially broken down by the proteolytic activity during soaking and germination, which made them more vulnerable to pepsin and pancreatin *in vitro* digestion. The functional properties including bulk density (0.17 ± 0.01 to 0.15 ± 0.01), true density (0.43 ± 0.06 to 0.30 ± 0.17), porosity (60.46 ± 1.23 to 50.00 ± 2.19), water solubility index (1.90 ± 0.69 to 5.67 ± 0.47), water absorption capacity (0.90 ± 0.02 to 1.51 ± 0.37), foaming capacity (12.67 ± 0.94 to 21.67 ± 0.94), foaming stability (0.98 ± 0.01 to 1.11 ± 0.03), oil absorption capacity (1.87 ± 0.03 to 2.07 ± 0.02) were found to be varied.

### 5.2 Cooking

Cooking can bring physical and nutritional alterations to BYM (Singh et al., 2022). Taking account of the nutritive value of BYM, a plethora of food products like bread, snacks, baby foods, fermented products, porridge, dumplings, fast foods, and millet powder can be made using different strategies like boiling, steaming, roasting, or baking (Shafie et al., 2023). The cooking time for BYM is observed to be less as compared to other millets, making it an ideal choice for quick meals (Bhatt et al., 2023). Also, from a sensory point of view, the mild, nutty flavor of BYM can complement different soups, stews, and salads. The enhancement in TPC can be attributed to the release of more bound phenolics after the hydrolysis of cellular constituents while carrying out high-temperature treatment (Siroha and Sandhu, 2017). The impact of cooking on protein digestibility could be due to protein denaturation and/or decreased resistance to enzyme attack (Orlien et al., 2023). However, in contrast to these results, a decrease in *in-vitro* protein and starch digestibility was observed after cooking millet grains (Kumar et al., 2020). The decrease in protein digestibility after cooking could be because of the aggregate formation through hydrophobic interactions caused by the orientation of tryptophan residues, thereby causing microstructural alterations in spherical protein bodies (1–2.5 mm) (Gulati et al., 2018; Cordelino et al., 2019). The alteration in *in-vitro* starch digestibility could be ascribed to the distorted amylose/amylopectin ratio and amylose-lipid complexes on account of the thermal treatments during millet processing (Bora et al., 2019). There was an increase in total dietary fiber in ready-to-cook BYM flakes, which would be because of the development of resistant starch during moist steaming and cooling cycles carried out during the processing (Patil et al., 2014). Cooking is also a feasible strategy to reduce antinutrients like phytic acid, which aids in improving the bioavailability of minerals such as iron and zinc and also *in-vitro* protein digestibility (Gowda et al., 2022). Therefore, soaking, followed by the cooking of BYM grains, can be identified as a pre-treatment to decrease the anti-nutritional factors and improve the nutrient bioavailability of food products.

### 5.3 Fermentation

Fermentation is an appropriate strategy in the preservation of foods, aids in the enhancement of flavor, vitamins, and minerals, and deduces the antinutrients present in food products (Sasi et al., 2022). Natural fermentation enhances the reduced sugars, TPC, antioxidant potential, mineral concentration, and other nutrients of foods via the synthesis of amino acids, vitamins, and proteins, and reduces the tannin and phytate content (Samtiya et al., 2020). Fermentation of BYM by *L. plantarum* has an additional advantage in terms of nutritional content as well as antioxidant activity and also increases the shelf life of BYM-based probiotic products (Sasi et al., 2025). The TPC was observed to be higher (198 ± 0.1 mg GAE/100g) as compared to the unfermented BYM (150.93 ± 1.2 mg GAE/100g) (Srivastava et al., 2021). Fermentation also resulted in an enhancement in mineral content like 28 ± 0.81 mg iron from 14.5 ± 0.01 mg; 29.1 ± 0.02 mg calcium from 23.14 ± 0.21 mg; 12.03 ± 0.13 mg zinc from 6.8 ± 0.21 mg; and 3 ± 0.15 mg folate from 2.37 ± 0.21 mg per 100g in BYM (Srivastava et al., 2021). The presence of dietary fiber in BYM provides health effects as it acts as a substrate for fermentation in the large intestine (colon) and produces SCFA like butyrate, propionate, and acetate. Among these SCFA, butyrate aids in the regeneration of the colonic mucosa, being a source of energy, hence reducing the risk of colon cancer and inflammatory bowel disease (Yang et al., 2024). Meanwhile, propionate and acetate enter the splenic circulation and are mobilized into the liver, where they have been shown to impede cholesterol synthesis by hepatic cells, contributing to the hypocholesterolemia effects of dietary fiber (Wang et al., 2024). Another study recognized eighteen volatile compounds, i.e., cyclohexasiloxane, dodecamethyl (7.25%), cycloheptasiloxane, tetradecamethyl (5.83%), benzene, 1,3-bis-1,1-dimethylethyl (5.66%), dodecane (5.53%), eicosane (4.62%), cyclooctasiloxane, hexadecamethyl (4.54%), and significant amounts of decanal, heptane, 2,4-dimethyl, benzene, 1-ethyl-3-methyl, and mesitylene (Sheela et al., 2021) in fermented BYM milk. Most of these compounds are classified as essential volatile components for human health as they possess antioxidant, anti-inflammatory, antimicrobial, anticancerous, hypocholesterolemic, lubricant, and hemolytic properties (reductase inhibitors) and are also known to influence the aroma of fermented products.

In a study, the tannin content was observed to be reduced by 14.99% in fermented BYM under optimized conditions (Srivastava et al., 2024). Fermentation is also observed to lower the trypsin and amylase inhibitory activities in millets (Oladosu et al., 2016). Overall, fermentation is a very resourceful and convenient technique, either alone or in combination with other processing methods, to synthesize functional BYM products.

## 6 Physical treatments

### 6.1 Irradiation

Gamma rays are a commonly used electromagnetic radiation among the physical mutagens for the treatment of food commodities on account of their high and uniform penetrating capacity (Wang, 2026). Gamma irradiation also safeguards foods against insect attack and spoilage by inhibiting enzymatic activities (Wani et al., 2021). Food and Agriculture Organization, the International Atomic Energy Agency, and World Health Organization agreed that the exposure of food commodities to a gamma radiation dose of up to 10-kilo gray does not alter their nutritional properties but instead modifies their physicochemical, functional, and antioxidant properties (Bangar et al., 2024). Gamma irradiation of 5-kilo grey has been observed to ameliorate the nutraceutical potential of BYM grass seed flour by reducing the swelling index (from 8.85 ± 0.35 to 6.35 ± 0.06), peak viscosity (from 1512.0 ± 0.1 to 1441.0 ± 34.0), setback viscosity (1913.5 ± 14.6 to 948.5 ± 14.5), foaming capacity (from 13.7 ± 2.41 to 10.41 ± 0.41), and foaming stability (from 34.0 ± 4.58 to 21.1 ± 10.56). Also, gamma irradiations were identified to enhance water and oil absorption potential, emulsion capacity, and antioxidant attributes (Chawla et al., 2009). BYM grass seed flour irradiated with 5-kilo gray exhibited an antioxidant activity of 14.68%. This high antioxidant content of irradiated BYM grass seed flour could be attributed to the occurrence of Maillard’s reaction between reducing sugars and amino acids synthesized in flour after irradiation, which resulted in the synthesis of compounds called melanoidins (Choi et al., 2009). BYM grass seed flour irradiated with 5-kilo gray doses showed a lipid peroxidation inhibition of 43.41%, which can be attributed to the possibility that gamma irradiation-induced dissociation generates C-O groups that form chelated complexes with metal ions and prevent lipids from oxidation (Verma et al., 2018). Additionally, Wani et al. (2022) demonstrated that the swelling power, pasting properties, and syneresis of BYM starch were all considerably altered by irradiation at 2.5 and 5 kGy.

The frequency and spectrum of chlorophyll mutations and the efficiency of a combination of ethyl methane sulfonate and gamma-ray irradiation with doses ranging from 500 to 1000 Gy were studied in BYM variety CO(Kv) (Ramesh et al., 2019). The spectrum of chlorophyll mutations was categorized into various types, viz., albina, chlorina, xantha, viridis, and strata. The mutation spectrum revealed that chlorina (0.58%) happened with the highest frequency, followed by xantha (0.55%), albino (0.28%), viridis (0.18%), and striata (0.11%). The study shows BYM will react well to physical and chemical mutagens like EMS and gamma-ray irradiation and offers scope for economic mutants with a higher yield.

### 6.2 Cold plasma treatment

Cold plasma therapy has come to light as a viable method in the search for cutting-edge and sustainable food processing technologies. It can improve grains’ nutritional and functional qualities without lowering their heat-labile components (Sarkar et al., 2023). Kaur et al. (2020) recognize cold plasma treatment as a non-thermal method. It is commonly defined as an ionized gas that is mostly made up of reactive nitrogen species (ROS), excited or ground-state electrons, energetic ions, ozone, and free radicals.

Multipin atmospheric cold plasma (MACP) was applied to dehulled and ground BYM for 10–30 min at varying power levels (10–30 kV) (Sharma et al., 2024). In particular, TPC (228.23–281.24 mg GAE/100 g) and flavonoids (172.76–245.31 mg quercetin equivalents/100 g) increased after 30 kV:30 min 1-methylcyclopropene treatment on BYM flour. Additionally, physicochemical parameters such as color, pH, moisture content, proximate composition, and shape changed.

According to the study (Charu et al., 2024), BYM is more sensitive than pearl millet to cold plasma treatment. Following cold plasma treatment, there was a rise in antioxidant activity (41.14%), fat (4.8 ± 0.15), TPC (47.10 ± 0.58), TFC (345.13 ± 0.34), and vital vitamins (B3, B9 (46%), all of which are critical for maintaining human health. Phytic acid was down 62%, and tannin concentration was significantly reduced (by 25%) in BYM. But it’s known that cold plasma treatment can help cause these increases in several ways, such as breaking down anti-nutrients like phytic acid, improving the release of nutrients from damaged cell structures, activating enzymes that help synthesize vitamins, lowering the number of microorganisms present, and helping to retain heat-sensitive vitamins because the treatment produces less heat. Ten chemicals quantified and found to increase were oleic acid, γ-tocopherol, 3-cyclohexene-1-methanol, α, α,4-trimethyl-acetate, n-hexadecanoic acid, 9,12-octadecadienoic acid, octadecanoic acid, raffinose, and β-Sitosterol. Reactive species found in cold plasma also modify the way starch works. Certain types of starch and plasma may have different effects from one another, and it may result in cross-linking, depolymerization, etching, and starch oxidation (Zhu, 2017).

**Tables 4**, **5** presents the impact of various processing methods on biochemical parameters of BYM and discussing the comparative effectiveness and practical feasibility of each processing methods. Existing technologies are required to be assessed thoroughly to identify the gaps and make them more dynamic and effective for BYM processing (Sharma S et al., 2022).

**Table 4:** Impact of various processing methods on biochemical parameters of BYM.

Sr. no.	Processing techniques	Parameters	Change in value	References
1	Dehulling or Decortication	Crude protein Crude Ash Digestible Crude protein Total Digestible Nutrient	9.19- 9.47 mg/10g 5.53-6.47 mg/10g 4.41- 4.67 mg/10g 79.98- 83.43 mg/10g	Sood et al., 2023
2	Soaking	TPC Total Antioxidant activity	54.5 mg of GAE/g 87.90% (DPPH scavenging)	Murugan et al., 2016
3	Germination	Antioxidant activity TPC Total Flavonoids	65.25 to 91.99% 28.01 to 54.99 mg GAE/100 g 23.45 to 42.56 mg RU/ g	Sharma et al., 2016
4	Cooking	Carbohydrate Fat Vitamin C Vitamin E Alkaloid Oxalate Fe		Rana et al., 2023
5	Fermentation	Antioxidant activity FRAP content TPC content Fe content Tannin	13.13% (% DPPH scavenging) by 13% by 3.2% up to 8.7% reduced by 14.97%	Srivastava et al., 2021
6	Cold Plasma	TPC content Flavonoid content	229.33–280.54 mg GAE/100 g 173.75–244.71 mg quercetin equivalents/100 g	Sharma et al., 2024
7	Gamma irradiation (5 kGy dose)	DPPH inhibition TPC content Inhibition of lipid peroxidation	1.68% to 14.67% 0.41 to 0.85 mg GAE/g 32.9–43.81%	Wani et al., 2021

**Table 5:** Comparison of various processing techniques applied to BYM and their practical feasibility.

Sl No.	Processing method	Reduction of antinutritional factors (ANFs)	Enhancement of bioactive compounds	Functional property modifications	References	Practical feasibility
1	Dehulling or decortication	Total Phytate ↓ Tannin ↓	--	LDL C ↓ VLDL ↓ Protein digestibility ↑ Glycemic index ↓	Goel et al., 2021 Ugare et al., 2014	Simple Low cost Highly scalable Limited to moderate nutritional enhancement
2	Soaking	Phytate ↓ (moderate)	Total polyphenol content (TPC) ↑ Total flavonoid content ↑	Antioxidant property ↑ Protein digestibility ↑	Sharma et al., 2016	Easy to implement at household and industrial level Cost effective Scalable with minimal infrastructure Moderate nutritional quality improvement
3	Germination	Total ANFs ↓ Phytate ↓ due to ↑ in phytase activity	γ-aminobutyric acid (GABA)↑ Free phenolic compounds ↑ Fe ↑; Zn ↑; Mg ↑; Ca ↑; Na ↑	Protein digestibility ↑ Varied functional properties, like bulk density, true density, porosity, water solubility index, foaming capacity, foaming stability of BYM flour	Dey et al., 2026 Sharma et al., 2016	Time dependent process Low cost Moderate scalability Soaking and germination amplify overall nutri-functional properties of BYM flour
4	Cooking (Thermal Processing)	Phytate ↓	TPC ↑ at high temp. Mineral bioavailability ↑ (eg. Fe ↑; Zn ↑; Mg ↑; Ca ↑; Na ↑)	Protein digestibility ↓ Starch digestibility ↓ Total Dietary Fibre (DF)↑	Orlien et al., 2023 Gowda et al., 2022 Kumar et al., 2020 Siroha and Sandhu, 2017	Highly scalable due to ↓ processing time Household and industrially feasible Soaking followed by cooking enhances nutritional quality through ↑ nutrient bioavailability
5	Fermentation	Tannin ↓ Phytate ↓	TPC ↑ Mineral concentration ↑ Folate ↑ DF fermentation leading to the ↑ production of Short Chain Fatty Acids (SCFA), eg: butyrate, propionate, acetate	Reduced sugar ↑ Antioxidant potential ↑ Trypsin inhibitory activities ↓ Volatile compound generation ↑ having immunomodulatory effect	Yang et al., 2024 Srivastava et al., 2021 Sheela et al., 2021 Samtiya et al., 2020 Srivastava et al., 2024 Oladosu et al., 2016	Moderate-highly scalable Requires controlled processing condition Combinatorial approach with other processing techniques enhances functionality of BYM
6	Irradiation	--	--	Swelling index ↓ Peak viscosity ↓ Setback viscosity ↓ Foaming stability ↓ Foaming capacity ↓ Water and oil absorption potential ↑ Emulsion capacity ↑ Antioxidant potential ↑ Lipid peroxidation ↓ Alternation in swelling power, pasting properties, and syneresis of BYM starch	Wani et al., 2022 Chawla et al., 2009 Choi et al., 2009	Industrially feasible Moderate operational cost Radiation safety concern Regulatory constraints
7	Cold plasma therapy, specifically MACP	Anti-nutrients like phytic acid ↓	TPC ↑ Flavonoids ↑ Heat sensitive vitamins like B3, B9 ↑ Oleate ↑ Tocopherol ↑ Raffinose ↑ β-sitosterol ↑	Antioxidant activity ↑ Reactive species induce modifications in starch digestibility and food matrix interactions	Sharma et al., 2024 Charu et al., 2024	Minimal thermal degradation, preserving heat sensitive nutrients Moderate scalability with emerging industrial potential Requires specialized equipment and technical expertise

## 7 Sensory attributes influencing acceptance

Important sensory characteristics like taste, fragrance, color, and overall mouthfeel have a significant impact on consumer acceptability of BYM. Studies on BYM-based products such as kheer, biscuits, and extruded snacks show high acceptability scores (>8 on a 9-point scale) for optimized formulations. However, higher levels of millet incorporation frequently introduce slight bitterness or cereal aftertaste, reducing consumer preference (Verma et al., 2015). Texture is another important attribute because the high dietary fiber content of BYM contributes to a coarse or gritty mouthfeel. Studies on ready-to-eat BYM snacks and bakery products similarly report that excessive millet inclusion leads to hardness and reduced sensory scores, confirming the importance of formulation balance (Goswami et al., 2015). When combined with ingredients like milk, jaggery, or spices, the aroma and flavor profiles often characterized as slightly earthy or nutty can significantly impact acceptance, as demonstrated in classic recipes like BYM kheer (Aundhkar et al., 2024).

## 8 Agronomic constraints

Because of their adaptability and storability, millets-often referred to as “famine reserves” are extremely robust crops that are ideal for India’s monsoon-dependent agriculture. BYM increases economic returns when grown for both food and fodder. Their contribution to sustainable development is highlighted by the fact that a 1% increase in output may result in a 0.65% decrease in poverty (Tiwari et al., 2023). A number of agronomic restrictions limit the yield and widespread use of BYM. In comparison to major cereals like *Oryza sativa* and *Triticum aestivum*, the crop typically shows lesser production potential, small grain size, and a high propensity for seed breaking, which causes harvest losses. It is quite vulnerable to weed competition because of its delayed beginning growth, especially in rainfed environments (Anuradha et al., 2025). Furthermore, the crop is vulnerable to waterlogging and inadequate drainage even if it can withstand drought. Yield optimization is further hampered by the lack of standardized agronomic procedures and the scarcity of high-yielding, disease-resistant cultivars. Harvesting efficiency is further decreased by problems including lodging, uneven maturity, and poor fit for mechanization (Goron and Raizada, 2015).

## 9 BYM residue: sustainable source of bioethanol production

The current trend of fluctuating oil prices, environmental degradation, and global warming has prompted major global consumers to dramatically boost the use of “green” fuels (Bashir et al., 2024). It is thought that turning millet agro-residues into reusable energy, like second-generation bioethanol, provides a sustainable closed-loop solution to problems related to waste management and energy recovery (Lamichhane et al., 2023). There are two phases involved in the production of bioethanol from lignocellulosic biomass: hydrolysis and fermentation (Tagade and Sawarkar, 2023). The presence of a high amount of cellulose, hemicellulose, and a lower mass proportion of lignin indicates that millet residues are suitable for slow pyrolysis-induced high biochar formation from the lignocellulosic biomass (Umakanth et al., 2024), and these millet remains can therefore be used to produce a lot of bio-oil. The high cellulose and hemicellulose percentages make C5 and C6 sugars accessible for bioconversion to bioethanol. However, it is crucial to pretreat the biomass to remove the lignin barrier from the plant cell wall and release the cellulose and hemicellulose for fermentation (Awoyale et al., 2021).

In a research study, the lignin content in BYM was observed to be 22.30%, while the cellulose content was 35.5% (Gairola et al., 2023). It was discovered that the hemicellulose content in finger millet, sorghum, and pearl millet straw was 32.5%, 30.33%, and 29.5%, respectively, but the amount in BYM was significantly higher, i.e., 33.17% (Gairola et al., 2022). The crude fiber content in BYM ranges from 4.9 to 6.1 g (12.6%) (Nazni and Devi., 2016). The crude ash content in BYM ranges from 4.50% to 6.47%, whereas in its husk it ranges from 0.63% to 1.03% (Sood et al., 2023).

From the point of view of a circular bioeconomy, this utilizable form can improve the economic value of millet-based production systems while converting the residue into renewable energy. Moreover, only a few reports have come regarding the structural characterization of BYM lignocellulose and how it influences the efficiency of bioprocessing. As there are still a lot of unanswered questions regarding the effective utilization of BYM residue into fermentable sugars, despite its potential. However, to increase the sugar yields, and ethanol productivity, various pretreatment techniques, the enzymatic systems, microbial fermentation strategies must be optimized combining the omics-based techniques with metabolic engineering of microbial strains (Saied et al., 2024).

Efforts should be taken on making energy efficient and low cost integrated biorefinery models for BYM residue, which enables the synthesis of bioethanol and other products like lignin-derived chemicals, organic acids, and biofertilizers, simultaneously. Further, evaluation of the environmental performance of BYM based bioethanol including quantifying the greenhouse gas emissions, energy balance, water footprint, and waste generation across the various stages of production. Integration of life cycle assessment with techno-economic analysis will be essential to find the optimal process configurations and make sure that the bioethanol production models will be both environmentally sustainable and economically viable.

## 10 Conclusion and future perspectives

**Figure 1:**
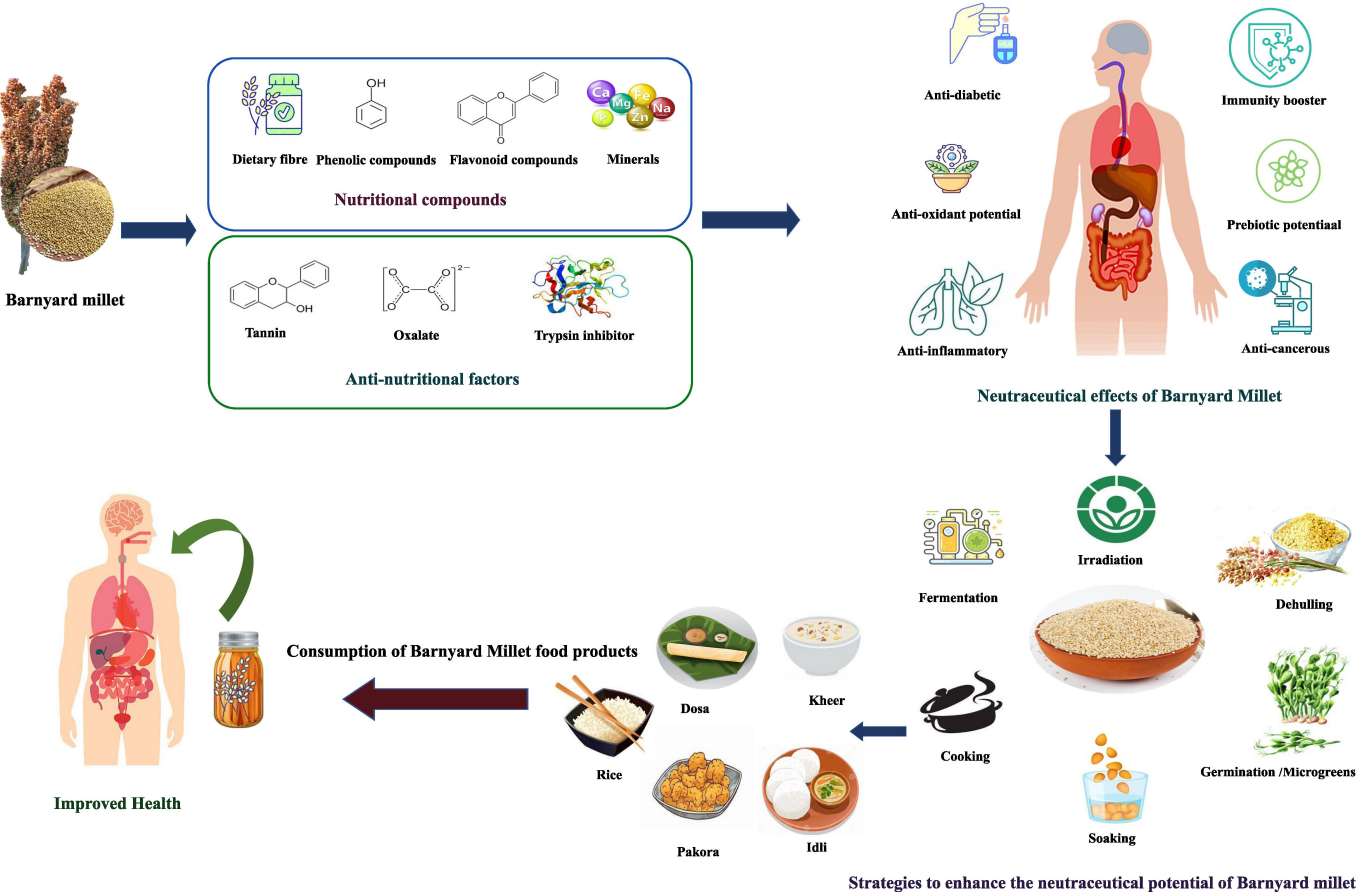
Components discussed in the review.

**Figure 2:**
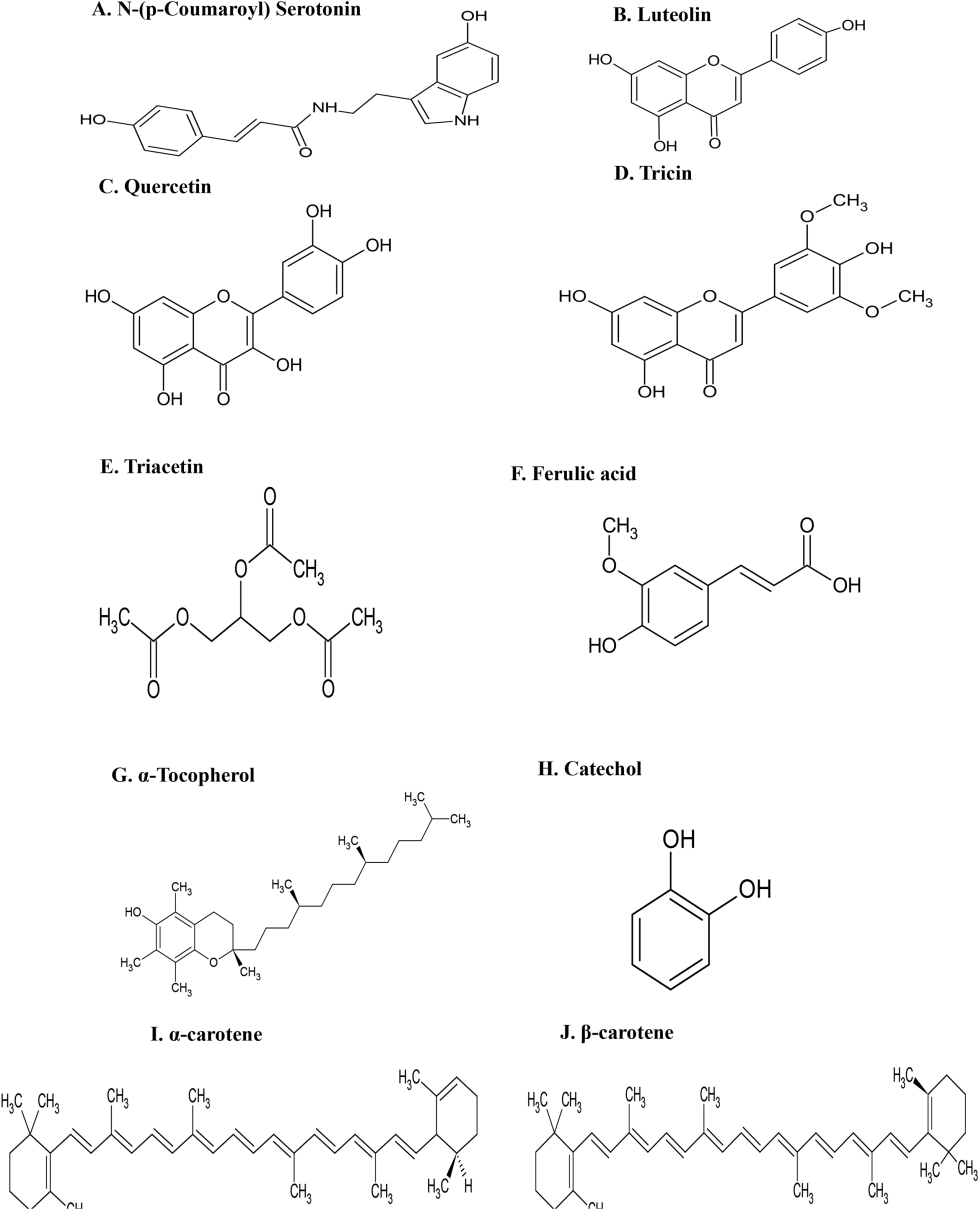
Representative chemical structures of the major antioxidants present in BYM. **(A)** N-(p-coumaroyl) serotonin **(B)** luteolin **(C)** quercetin **(D)** tricin **(E)** triacetin **(F)** ferulic acid **(G)** α-tocopherol **(H)** catechol **(I)** α-carotene **(J)** β-carotene.

As researchers and global citizens, we anticipate a sustainable time ahead with food and nutritional security, the world needs many more stress-tolerant, resource-efficient, and nutrient-diverse cereals crops. The objective of this review article is to educate people about the health benefits of millets as whole grains and value-added ready-to-cook and ready-to-eat products. Millets, especially BYM are undoubtedly superior to staple cereal crops like rice and wheat owing to their remarkable phytochemical composition including phenolic compounds, dietary fiber, flavonoids and phytosterols. These dietary components in BYM aid to cope with various diseases on account of their prebiotic properties, antioxidative mechanisms, anti-inflammatory potential, hypoglycemic profiles and anticancerous attributes (**Figure 1**). Therefore, involving millet foods especially BYM in our daily diet is an excellent approach. However, BYM harbors certain anti-nutritional factors like α-amylase inhibitor, tannins, phytic acid and trypsin inhibitor which interrupt the bioavailability of minerals and other nutrients (**Figure 2**). Hence, valorization techniques such as soaking, roasting, germination or fermentation are carried out to decrease the number of antinutrients and also to enhance the bio accessibility and bioavailability of micronutrients. Also, despite its nutritional benefits, BYM remains to be an under-utilized cereal because its cultivation is distinctly concentrated in states like Uttarakhand and Tamil Nadu but less productivity is a challenging factor.

However, existing strategies have to be re-evaluated and the identification of gaps followed by significant efforts would enhance the productivity of BYM. Deeper mechanistic insights about the molecular pathways and bioavailability mechanisms need to be explored, especially at the molecular and metabolic levels. Furthermore, more studies should be conducted about BYM’s shelf life and interactions with biological systems under various processing conditions. Though the initial results are fulfilling, further confirmation using more *in-vivo* animal models and human clinical trials is essential. Future studies should emphasize on establishing safety profiles, efficacy, and dose-response correlations of BYM. Further, to enhance specific traits, such as nutritional composition, improved bioavailability, and reduce the anti-nutritional factors, strategic breeding methods, such as marker-assisted selection and genome-editing techniques like CRISPR-Cas systems, should be used in future. The scale up feasibility of processing methods, cost-effectiveness of these methods, regulatory permissions, sensory attributes and consumer acceptability are some of the challenges that still exist despite technical breakthroughs.
